# Prevalence and risk factors of chronic obstructive pulmonary disease in Anhui Province, China: a population-based survey

**DOI:** 10.1186/s12890-019-0864-0

**Published:** 2019-05-29

**Authors:** Zhenqiu Zha, Ruixue Leng, Wei Xu, Heling Bao, Yeji Chen, Liwen Fang, Zhirong Liu, Dongqing Ye

**Affiliations:** 1 Department of Epidemiology and Biostatistics, School of Public Health, Anhui Medical University, Hefei, 230032 Anhui China; 2 Anhui Provincial Center for Disease Control and Prevention, Hefei, 230601 Anhui China; 3 National Center for Chronic and Non-Communicable Disease Control and Prevention, Chinese Center for Disease Control and Prevention, Beijing, China; 4 Clinic Medical College of Anhui Medical University, Hefei, Anhui China

**Keywords:** Chronic obstructive pulmonary disease, Cross-sectional survey, Prevalence, Risk factors, Respiratory function tests

## Abstract

**Background:**

The prevalence of chronic obstructive pulmonary disease (COPD) in Anhui Province of eastern China remain uncertain. The present study provides the first estimate of the prevalence and risk factors of COPD in Anhui.

**Methods:**

A population-based survey was conducted in a representative sample of population aged 40 years or older in 2015. COPD was diagnosed based on 2017 Global Initiative for Chronic Obstructive Lung Disease (GOLD) criteria.

**Results:**

A total of 2770 participants had reliable post-bronchodilator results and were included in the final analysis. The overall prevalence of COPD was 9.8% (95% CI: 8.2, 11.7). Prevalence was higher in men (14.8, 95% CI: 12.6, 17.2) than it was in women (5.2, 95% CI: 3.1, 8.7). Among adults with COPD, 45.0% (95% CI: 39.1, 51.0) had moderate or severe disease (GOLD stage II-IV), 0.7% (95% CI: 0.2, 2.9) reported that they had a previous pulmonary function test, and only 0.4% (95% CI: 0.1, 2.6) knew their diagnosis of COPD. Risk factors for COPD included older age (OR 1.06, 95% CI: 1.04, 1.08), male sex (OR 2.01, 95% CI: 1.22, 3.33), current smoking status (OR 2.63, 95% CI: 1.86, 3.73), primary school or lower education (OR 1.61, 95% CI: 1.12, 2.31), family history of lung disease (OR 1.50, 95% CI: 1.17, 1.93), and indoor exposure to coal for cooking or heating (OR 1.55, 95% CI: 1.11, 2.15). In addition, people in north region has a significantly higher risk for developing COPD than people in south region of Anhui (OR 1.98, 95% CI:1.44, 2.71).

**Conclusions:**

COPD is prevalent in Anhui and the prevalence is highest in north region. Strategies aiming at prevention, early detection and treatment of COPD are urgently needed to reduce COPD-related burden.

**Electronic supplementary material:**

The online version of this article (10.1186/s12890-019-0864-0) contains supplementary material, which is available to authorized users.

## Background

Chronic obstructive pulmonary disease (COPD) is characterized by progressive airflow obstruction that is only partly reversible, and causes a worldwide public health problem [1]. About 90% of deaths caused by COPD occurred in low-income and middle-income countries [2, 3]. In 2016, it was the fifth leading cause of death in China, the largest developing country [4]. However, over the past decade, few studies had examined COPD prevalence based on post-bronchodilator test in China. A reliable survey of spirometry-defined COPD in China was conducted during 2002–2004 among 20,245 adults aged 40 years or older and it observed an overall prevalence of 8.2% [5]. More recently, two nationwide estimations indicated that the prevalence of COPD among people aged 40 years or older increased rapidly to approximately 13.6% during 2014–2015 [6, 7].

Available evidence has indicated that COPD prevalence could substantially vary across different regions of China, possibly because of their different economic levels, diverse lifestyles, and various population demographic patterns [5, 6]. The population of residents in Anhui Province of eastern China was approximately 60 million, and the proportion of people aged 40 years or older was 44.2% according to the 2010 Population Census [8]. Anhui province has undergone rapid economic development and significant changes in lifestyle in the past decades, and its people has seen a substantial increase in life expectancy. Moreover, the proportion of urban population of Anhui increased from 30.7% in 2002 to 50.5% in 2015 [8, 9]. Given the high prevalence of tobacco smoking in Chinese men and COPD is common but preventable disease, assessment of the current burden of COPD in Anhui Province is thus urgent for the development of region-specific public health policy and better allocation of health-care resources. However, a recent systematic review conducted by Fang et al. has indicated that no data is available for COPD prevalence in general adults of Anhui [6]. Whether smoking and other risk factors are important in determining the magnitude of prevalence is also uncertain. Apart from these, Anhui possesses special geographic location in eastern China. The Qinling Mountains-Huaihe River Line crosses the north of Anhui province. For a long time, China is roughly divided into two regions (south and north) according to the geographic boundary. It has been well recognized that the two sides of the line have substantially deferent geographical features, natural condition, culture and climate. Furthermore, The Yangtze River (the largest Asian river) also flows through the south of Anhui. The two lines thus divide Anhui into three regions, north, central and south. It might be very worth to investigate the prevalence of COPD across the three regions in one province. The present study attempted to fill this knowledge gap and provide the first estimation of the prevalence and risk factors of spirometry-defined COPD in Anhui.

## Methods

### Study design and participants

**Fig. 1:**
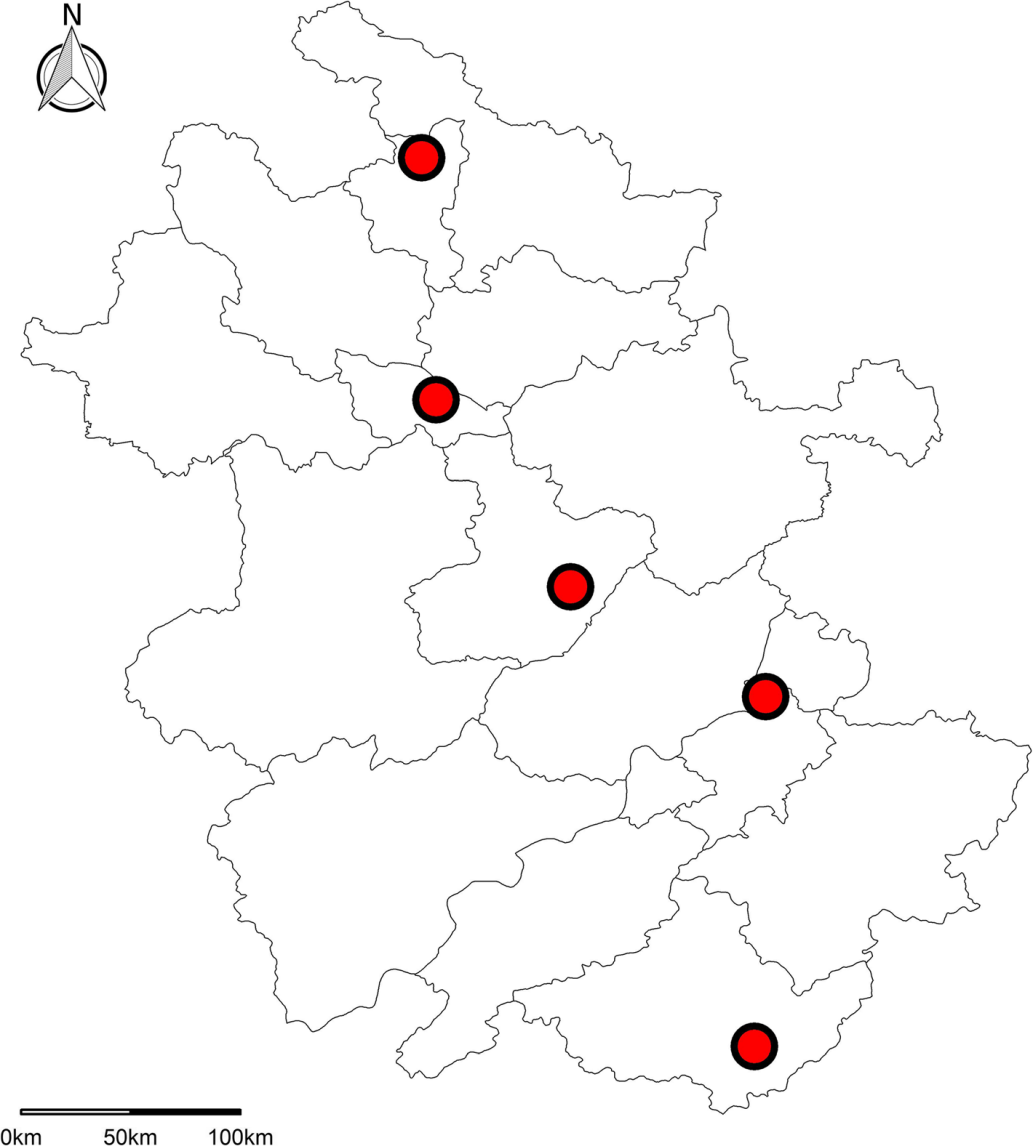
Locations of disease surveillance points (DSPs) used in the survey in Anhui Province of China

As a part of national estimation for COPD, the cross-sectional study in Anhui was also conducted with using the integrated national disease surveillance point (DSP) system from the Chinese Center for Disease Control and Prevention [6, 10, 11]. A complex, multistage, and probability sampling method was used to enroll a representative sample of adults aged 40 years or older in Anhui Province between Jan 1, 2015, and Jun 30, 2015. The first stage of sampling was stratified based on urbanization level (high or low). Within each stratum, at least two DSPs were randomly selected with probability proportional to the population size of the stratum. A total of 5 DSPs (Fig. 1) covering around 5% of the population in Anhui were selected. At each DSP, three sub-districts/townships were randomly chosen in the stage. Two neighboring communities/villages were then randomly selected within each sub-district/township. One group of villagers with at least 150 households was randomly chosen within each community/village. Finally, 100 households within each group of villagers were randomly chosen, and one family member aged at least 40 years old was selected randomly from each household by a Kish selection table [6]. The overall response rate defined based on the American Association for Public Opinion research was 99.9% in this study (Additional file 1: Table S1) [12].

The inclusion and exclusion criteria for participants in this study was based on the criteria applied in the nationwide prevalence estimation [6]. The study was approved by the Ethics Review Committees of Anhui Medical University and Anhui Provincial Center for Disease Control and Prevention. All subjects granted written informed consent to participate.

### Measures

All subjects who fulfilled above selection criteria were invited to attend an interview. Trained staff from local community clinics or health stations asked the participants questions based on a standardized questionnaire [6]. Subjects who reported never smoking were classified as never smokers. Those who reported current smoking at enrolment were classified as current smokers. Subjects who had stopped smoking prior to inclusion were classified as former smokers. We used qualitative levels (yes or no) for evaluating family (parental) history of respiratory. The term included the following lung diseases: asthma, chronic bronchitis, pulmonary emphysema, COPD, pulmonary heart disease, bronchiectasis, tuberculosis, rhinitis and lung cancer. Spirometry was tested following recommendations by the American Thoracic Society and was done by using the same brand of spirometer (MasterScreen Pneumo, Jaeger, Germany) [6, 13]. Prebronchodilator and post-bronchodilator forced expiratory volume in 1 s (FEV_1_), and forced vital capacity (FVC) were measured. These tests also included the modified Medical Research Council (mMRC) dyspnoea score and the Global Initiative for Chronic Obstructive Lung Disease (GOLD) 2017 ABCD assessment tool [1, 6]. The detailed information on these procedures (including definition of other COPD-specific risk factors, full details of the spirometry method used, and a quality grade method for spirometry results) is described in the nationwide prevalence study [6]. In this study, 99.1 and 98.3% of prebronchodilator and post-bronchodilator tests were considered as grade C or higher (acceptable grades), respectively.

In current study, COPD was defined as a post-bronchodilator FEV_1_:FVC ratio less than 70% based on 2017 GOLD guidelines [1]. The degree of obstruction was classified according to GOLD staging criteria [6, 7]. We calculated predicted values of FEV_1_ for normal lung function and the lower limited normal (LLN) of FEV_1_:FVC based on a nationwide study of reference values (available for 40-81 years in this study) for spirometry in the Chinese population [14, 15]. The LLN of Chinese reference values was used to define COPD in a sensitivity analysis [7, 14]. Other outcomes of our study included the awareness rate and treatment rate as applied elsewhere [6].

### Statistical analysis

The standardized prevalence was estimated in the overall population and in several subgroups. The prevalence was calculated using weights to represent the adult population aged 40 years or older in Anhui. The weights were calculated based on the study sampling scheme and data from the 2010 Population Census in Anhui [8]. These weights may account for several features of the study, including non-response, oversampling for several demographic differences between the sample and the total population of Anhui. Unweighted estimations were used for analyses of COPD severity in view of only COPD patients were included [6]. The analysis used all participants for whom the variables of interest were available. We did not impute missing data in this study [7, 16]. The statistical significance of difference was assessed by the χ^2^ test. Multivariable logistic regression was used to investigate potential risk factors for COPD. The calculation method for population-attributable fraction (PAF) was described by Graubard et al [17]. The current article follows the Strengthening the Reporting of Observational Studies in Epidemiology (STROBE) Statement for cross-sectional studies [18]. All statistical analyses conducted in 2018 were performed using Stata version 14.2 (Stata Corp, College Station, TX, USA). All statistical tests were 2-sided, and a *P* value less than 0.05 was considered statistically significant. Exact *P* values were provided when it was ≥0.001 in all conditions.

## Results

### Characteristics of study subjects

**Fig. 2:**
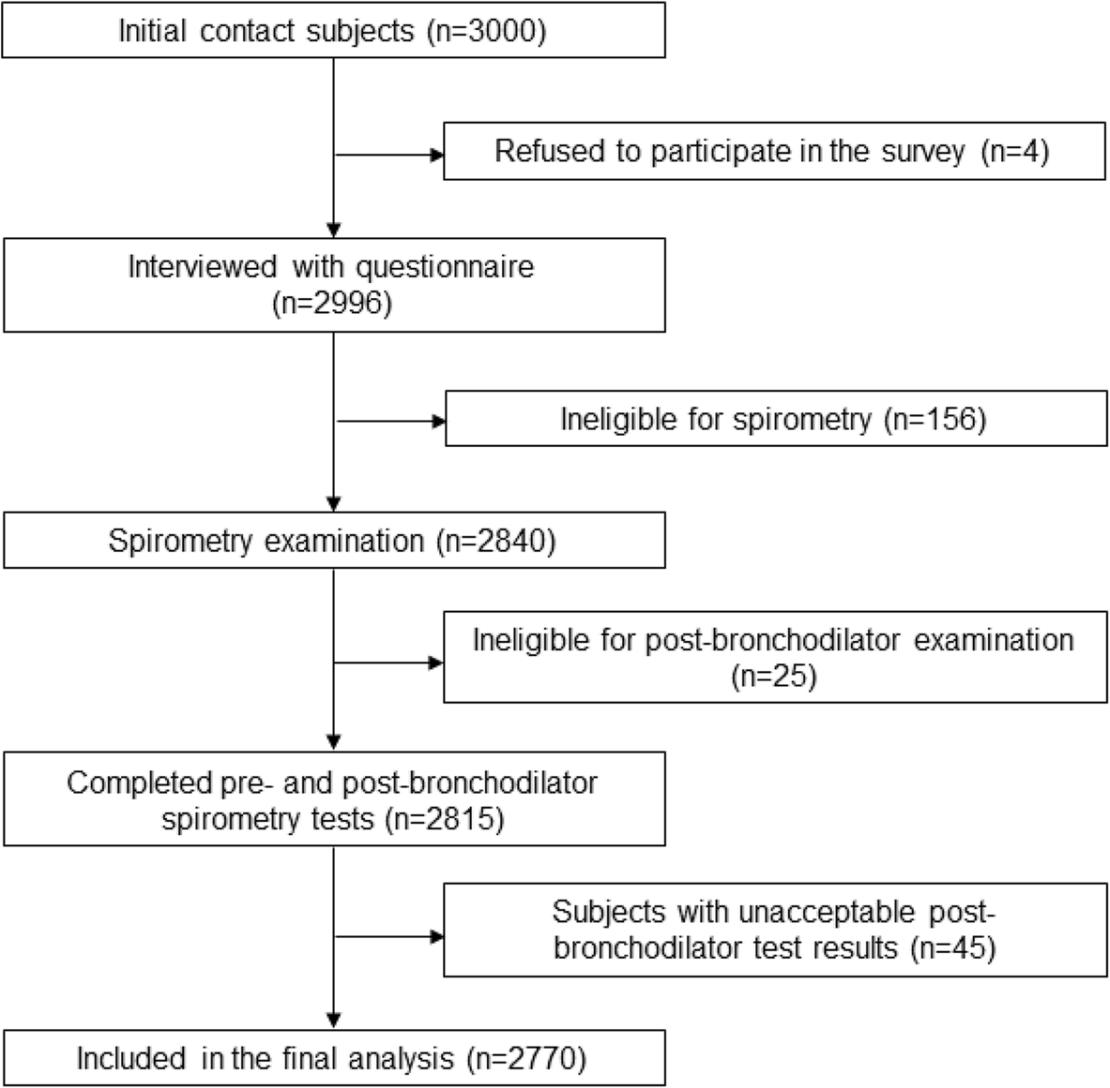
Flow of participants through the study

A total of 3000 subjects were invited to participate in the survey, of whom 2996 (99.9%) were interviewed. 2770 participants had acceptable postbronchodilator spirometry tests (grade A, B, or C) and were thus included in the final analyses (Fig. 2). The characteristics of subjects excluded and included are shown in the Additional file 1: Table S2. The cigarette smoking status was similar between two groups. However, subjects included in the analyses were younger, and had a higher proportion of females than those excluded. The weighted estimations of general characteristics of the 2770 individuals are presented in Table 1. Their mean age was 53.8 years (SD:10.5), and the mean value of their body-mass index (BMI) was 25.0 kg/m^2^ (SD: 3.3). The proportion of females were 51.8%. Regarding smoking status, 780 (28.5, 95% CI: 24.2, 33.2) were current smokers, that was 756 (57.3, 95% CI: 51.5, 62.9) of the men and 24 (1.8, 95% CI: 0.9, 3.8) of the women. The weighted proportions for other demographic characteristics and exposures of study participants are included in Table 1.

**Table 1:** General characteristics of study subjects from the population aged 40 years or older in Anhui

	Total (*n* = 2770)^a^	Men (*n* = 1362)^a^	Women (*n* = 1408)^a^
Age (years)	53.8 (10.5)	53.6 (10.4)	53.9 (10.5)
Body-mass index (kg/m^2^)	25.0 (3.3)	24.9 (3.2)	25.1 (3.4)
Residence			
Urban	1668/2770 (59.6% [35.5, 79.8])	770/1362 (60.0% [35.3, 80.4])	898/1408 (59.3% [35.5, 79.4])
Rural	1102/2770 (40.4% [20.2, 64.5])	592/1362 (40.0% [19.6, 64.7])	510/1408 (40.7% [20.6, 64.5])
Educational level			
Primary school or lower	1690/2769 (58.4% [47.5, 68.5])	668/1361 (42.8% [31.0, 55.5])	1022/1408 (72.8% [60.9, 82.2])
Secondary school	774/2769 (30.5% [23.7, 38.2])	497/1361 (41.2% [28.6, 55.1])	277/1408 (20.5% [15.4, 26.8])
Higher or further education	305/2769 (11.1% [3.6, 29.6])	196/1361 (15.9% [6.0, 36.0])	109/1408 (6.7% [1.5, 25.2])
Smoking status			
Never smoker	1758/2769 (63.8% [55.0, 71.7])	386/1361 (27.5% [17.7, 40.1])	1372/1408 (97.4% [95.0, 98.6])
Former smoker	231/2769 (7.7% [4.5, 13.0])	219/1361 (15.2% [10.0, 22.4])	12/1408 (0.8% [0.1, 5.0])
Current smoker	780/2769 (28.5% [24.2, 33.2])	756/1361 (57.3% [51.5, 62.9])	24/1408 (1.8% [0.9, 3.8])
Smoking exposure (pack-years)			
Pack-years = 0	1754/2718 (64.9% [55.1,73.5])	385/1317 (28.4% [17.8,42.0])	1369/1401 (97.8% [94.7,99.1])
0 < pack-years< 15	269/2718 (11.2% [7.8,15.9])	251/1317 (22.1% [14.4,32.5])	18/1401 (1.4% [0.7,3.0])
15 ≤ pack-years< 30	297/2718 (10.7% [6.8,16.6])	292/1317 (22.5% [15.6,31.2])	5/1401 (0.2% [0.0,0.9])
Pack-years≥30	398/2718 (13.1% [9.4,18.1])	389/1317 (27.0% [21.4,33.4])	9/1401 (0.6% [0.2,1.9])
Risk factors for COPD			
Hospital admission due to severe pulmonary disease in childhood	53/2769 (2.1% [1.2, 3.6])	26/1361 (1.7% [0.6, 5.0])	27/1408 (2.4% [1.7, 3.4])
Indoor exposure to biomass for cooking or heating	961/2769 (40.8% [26.1, 57.4])	489/1361 (37.5% [24.5, 52.7])	472/1408 (43.9% [27.4, 61.9])
Indoor exposure to coal for cooking or heating	518/2769 (28.9% [5.4, 74.3])	250/1361 (28.9% [5.6, 73.8])	268/1408 (28.9% [5.3, 74.9])
Exposure to dust or chemicals in the workplace	1152/2769 (43.5% [34.3, 53.2])	640/1361 (47.5% [35.9, 59.3])	512/1408 (39.9% [30.7, 49.8])
History of tuberculosis	46/2769 (1.3% [0.4, 4.4])	37/1361 (2.0% [0.5, 8.3])	9/1408 (0.6% [0.3, 1.3])
Spirometry			
Pre-bronchodilator FEV_1_	2.69 (0.71)	3.10 (0.71)	2.30 (0.45)
Post-bronchodilator FEV_1_	2.75 (0.72)	3.17 (0.72)	2.36 (0.45)
Pre-bronchodilator FVC	3.50 (0.89)	4.09 (0.80)	2.95 (0.57)
Post-bronchodilator FVC	3.51 (0.89)	4.11 (0.80)	2.96 (0.57)
Pre-bronchodilator FEV_1_: FVC	77.0% (7.7)	75.6% (8.6)	78.2% (6.5)
Post-bronchodilator FEV_1_: FVC	78.6% (7.6)	77.0% (8.6)	80.0% (6.3)

^a^Weighted estimations were used. Data are number exposed/total participants (%, 95 CI) or mean (SD)

*COPD* chronic obstructive pulmonary disease, *FEV*_*1*_ forced expiratory volume in 1 s, *FVC* forced vital capacity

### Prevalence of COPD and severity strata

A total of 272 subjects in this study were diagnosed with COPD based on 2017 GOLD guidelines. The overall standardized prevalence in people aged 40 years or older in Anhui was 9.8% (95% CI: 8.2, 11.7). The LLN defined prevalence was higher than the prevalence defined by GOLD (Table 2). The prevalence of COPD was higher in males (14.8, 95% CI: 12.6, 17.2) than it was in females (5.2, 95% CI: 3.1, 8.7; *P* = 0.006 for sex difference). Regarding smoking status, the overall prevalence was highest in current smokers (18.1, 95% CI: 11.7, 26.9) and lowest in never smokers (5.6, 95% CI: 3.8, 8.3) (Table 3). The prevalence of COPD in Anhui increased significantly with increments of pack-year (Table 3). The age-specific prevalence of COPD rose with increasing age in overall population and in male subjects (*P* < 0.05). Regarding region-specific patterns, the prevalence was 7.8% (95% CI: 5.8, 10.3) in south, was 10.7% (95% CI: 9.7, 11.8) in central and was 10.8% (95% CI: 6.5, 17.5) in north of Anhui.

**Table 2:** Age-specific prevalence of COPD in the general Anhui Chinese population

	Overall		Men		Women	
	No. of Participants	95% CI	No. of Participants	95% CI	No. of Participants	95% CI
Overall (crude/GOLD)	2770	9.8 (8.8, 11.0)	1362	15.4 (13.6, 17.4)	1408	4.4 (3.4, 5.6)
Overall (weighted/GOLD)	2770	9.8 (8.2, 11.7)	1362	14.8 (12.6, 17.2)	1408	5.2 (3.1, 8.7)
Overall (weighted/LLN)^a^	2745	12.2 (8.4, 17.3)	1349	16.6 (12.2, 22.1)	1396	8.1 (4.2, 15.1)
Age group (crude/GOLD)						
40–49 years	1047	3.9 (2.9, 5.3)	480	5.2 (3.5, 7.6)	567	2.8 (1.7, 4.6)
50–59 years	788	8.1 (6.4, 10.2)	335	11.9 (8.9, 15.9)	453	5.3 (3.6, 7.8)
60–69 years	635	15.9 (13.3, 19.0)	365	24.4 (20.2, 29.1)	270	4.4 (2.5, 7.7)
≥70 years	300	22.0 (17.7, 27.1)	182	30.8 (24.5, 37.9)	118	8.5 (4.6, 15.1)
*P* value for trend		< 0.001		< 0.001		0.012
Age group (weighted/GOLD)^a^						
40–49 years	1047	5.1 (3.2, 8.0)	480	5.9 (3.7, 9.5)	567	4.4 (1.6, 11.4)
50–59 years	788	9.0 (4.9, 16.0)	335	12.3 (5.3, 25.8)	453	5.6 (3.7, 8.4)
60–69 years	635	17.4 (10.5, 27.6)	365	30.1 (18.1, 45.7)	270	4.9 (1.8, 13.0)
≥70 years	300	19.9 (13.3, 28.6)	182	36.5 (18.5, 59.4)	118	8.3 (3.3, 19.6)
*P* value for trend		0.006		0.011		0.328
Age group (weighted/LLN)^a^						
40–49 years	1047	10.9 (6.7, 17.4)	480	12.0 (7.5, 18.8)	567	9.9 (4.7, 19.7)
50–59 years	788	11.0 (6.1, 19.2)	335	15.0 (6.6, 30.4)	453	7.0 (5.3, 9.3)
60–69 years	635	14.6 (9.7, 21.5)	365	24.9 (18.0, 33.4)	270	4.5 (1.2, 15.9)
≥70 years	275	16.4 (10.1, 25.6)	169	28.0 (13.9, 48.2)	106	7.8 (3.6, 16.2)
*P* value for trend		0.036		0.006		0.315

^a^The LLN defined COPD is only available for participants aged 40-81 years

COPD, chronic obstructive pulmonary disease; GOLD, the Global Initiative for Chronic Obstructive Lung Disease; LLN, lower limits of normal

**Table 3:** Prevalence of COPD in the general Anhui Chinese population aged 40 years or older, by demographic, and social characteristics

	Overall		Men		Women	
	Cases/total (n/N)	Prevalence of COPD (95% CI)	Cases/total (n/N)	Prevalence of COPD (95% CI)	Cases/total (n/N)	Prevalence of COPD (95% CI)
Residence						
Urban	156/1668	10.1 (6.4, 15.6)	116/770	15.3 (9.3, 24.4)	40/898	5.2 (2.5, 10.4)
Rural	116/1102	9.4 (6.1, 14.2)	94/592	13.8 (8.3, 22.2)	22/510	5.3 (3.7, 7.5)
*P* value		0.790		0.755		0.905
Educational level						
Primary school or lower	177/1690	11.0 (9.1, 13.3)	126/668	19.8 (16.5, 23.5)	51/1022	6.3 (3.7, 10.5)
Secondary school	69/774	7.9 (5.8, 10.6)	62/497	11.1 (8.6, 14.2)	7/277	1.9 (0.5, 6.6)
Higher or further education	25/305	8.0 (5.2, 12.1)	21/196	9.8 (6.8, 14.1)	4/109	4.1 (1.3, 12.0)
*P* value for trend		0.030		0.001		0.075
Smoking status						
Never smoker	92/1758	5.6 (3.8, 8.3)	34/386	7.9 (6.0, 10.3)	58/1372	5.0 (2.8, 9.0)
Former smoker	41/231	13.0 (2.9, 42.2)	41/219	13.7 (2.9, 45.3)	0/12	0
Current smoker	138/780	18.1 (11.7, 26.9)	134/756	18.1 (11.4, 27.4)	4/24	17.8 (5.9, 42.8)
*P* value		0.037		0.230		0.184
Smoking exposure (pack-years)						
Pack-years = 0	92/1754	5.6 (3.8, 8.3)	34/385	7.9 (6.0,10.3)	58/1369	5.0 (2.8,9.0)
0 < pack-years< 15	31/269	12.6 (6.4,23.5)	30/251	13.1 (6.2–25.5)	1/18	6.2 (0.5,48.8)
15 ≤ pack-years< 30	45/297	10.9 (4.7,23.3)	45/292	11.0 (4.7–23.6)	0/5	0
Pack-years≥30	97/398	26.1 (18.1,36.0)	94/389	25.7 (18.5,34.6)	3/9	38.6 (7.3,83.4)
*P* value for trend		0.001		< 0.001		0.116
Hospital admission for severe pulmonary disease in childhood						
Yes	3/53	4.0 (0.6, 23.3)	2/26	5.3 (0.2, 59.8)	1/27	3.2 (0.3, 29.8)
No	268/2716	9.9 (8.4, 11.5)	207/1335	14.8 (13.0, 16.8)	61/1381	5.3 (3.1, 8.8)
*P* value		0.189		0.333		0.552
Indoor exposure to biomass for cooking or heating						
Yes	110/961	11.0 (9.5, 12.7)	90/489	18.7 (15.9, 21.8)	20/472	5.0 (3.5, 7.1)
No	161/1808	8.9 (6.7, 11.6)	119/872	12.2 (10.5, 14.0)	42/936	5.4 (2.5, 11.4)
*P* value		0.090		0.007		0.739
Indoor exposure to coal for cooking or heating						
Yes	58/518	11.5 (9.8, 13.4)	49/250	19.3 (16.9, 21.9)	9/268	4.2 (1.8, 9.5)
No	213/2251	9.0 (7.1, 11.5)	160/1111	12.7 (10.8, 14.9)	53/1140	5.6 (3.2, 9.9)
*P* value		0.075		0.006		0.352
Exposure to dust or chemicals in the workplace						
Yes	120/1152	9.6 (7.5, 12.3)	101/640	14.5 (11.4, 18.1)	19/512	4.9 (2.7, 8.7)
No	151/1617	9.9 (7.4, 13.1)	108/721	14.7 (11.8, 18.3)	43/896	5.5 (2.9, 10.2)
*P* value		0.850		0.873		0.606
Body-mass index (kg/m^2^)						
< 18.5	6/42	9.5 (1.8, 37.9)	5/23	17.8 (5.9, 43.1)	1/19	2.7 (0.1, 44.9)
18.5–23.9	137/1173	12.4 (7.4, 19.9)	114/602	20.0 (12.5, 30.4)	23/571	5.1 (2.1, 12.0)
24.0–27.9	99/1114	8.9 (7.2, 10.9)	70/547	10.9 (8.3, 14.2)	29/567	6.9 (3.6, 12.6)
≥28.0	30/441	6.3 (5.4, 7.4)	21/190	11.5 (7.5, 17.1)	9/251	2.5 (1.0, 5.9)
*P* value for trend		0.094		0.044		0.135

*COPD* chronic obstructive pulmonary disease

Additional file 1 Table S3 presents the severity based on GOLD stage in patients with COPD. Patients with GOLD stage III and IV were combined because of small sample size. Among these individuals, 55.0% (95% CI: 49.0, 60.9) were GOLD stage I, 35.3% (95% CI: 29.8, 41.3) were GOLD stage II, and only 9.7% (95% CI: 6.6, 13.8) were GOLD stage III/IV. Among the 270 subjects who were diagnosed with COPD and had complete information on mMRC score, 15.2% had an mMRC score of 2 or higher, and 93.7% were classified as “A” according to the GOLD ABCD assessment tool (Additional file 1: Table S4).

### Respiratory symptoms and awareness of COPD

The proportion of patients with COPD with respiratory symptoms is presented in the Additional file 1: Table S5. Overall, 42.8% (95% CI: 37.0, 48.8) of subjects with COPD had at least one respiratory symptom, and 15.1% (95% CI: 11.3, 19.9) had chronic cough and phlegm. The proportion of respiratory symptoms in patients with COPD increased with GOLD severity strata (*P* < 0.05). Additional file 1: Table S6 presents the proportion of comorbidities in patients with COPD. Of all individuals with COPD, 25.8% (95% CI: 20.9, 31.4) also lived with hypertension. It should be noted that 5.9% (95% CI: 3.6, 9.4) of subjects with COPD had a history of exacerbation in the past year, and only 1.8% (95% CI: 0.7, 4.4) had a history of admission to hospital for COPD (Additional file 1: Table S6). Only 0.4% (95% CI: 0.1, 2.6) of subjects diagnosed with COPD were aware of their COPD condition, 0.7% (95% CI: 0.2, 2.9) had ever been examined by spirometry prior to the investigation, and 7.7% (95% CI: 5.1, 11.6) were treated for this disease (Additional file 1: Table S7).

### Risk factors of COPD

Univariate analysis demonstrated that smoking behavior (current/former) and lower education level (primary school or lower) were correlated significantly with higher COPD prevalence in overall population. It should be noted that exposure to biomass/coal for cooking or heating was also marginally associated with the estimated prevalence in Anhui (Table 3). In subgroup analysis of sex, lower educational degree and indoor air pollution were positively correlated with COPD proportion in males only. Using adjusted analysis, the logistic model showed that older age at survey, men, current smoking status, lower degree of education, indoor air pollution (coal for cooking/heating), and parental history of respiratory disorders were correlated with increased COPD risk (Table 4). In addition, normal BMI (18.5–23.9 kg/m^2^) was associated with the highest COPD risk in overall population as well as in male subjects (Table 4). We further estimated risks of COPD in the three regions of Anhui province. Compare to south, both central and north had higher risk for developing COPD (north: OR = 1.45, 95% CI:1.10, 1.90, *P* = 0.023; central: OR = 1.42, 95% CI:1.10, 1.84, *P* = 0.023). However, only north region had a significant difference with south after adjusting sex and age (north: OR = 1.86, 95% CI:1.46, 2.37, *P* = 0.004; central: OR = 1.12, 95% CI:0.60, 2.07, *P* = 0.605). The association was still robust after full adjustments (including all factors listed in Table 4). The OR was 1.98 (95% CI:1.44, 2.71, *P* = 0.006) in north and was 1.26 (95% CI:0.65, 2.45, *P* = 0.344) in central of Anhui. In this study, the overall PAFs for current smoking and exposure to indoor air pollution (coal for cooking/heating) were 32.8 and 12.1%, respectively (Additional file 1: Table S8).

**Table 4:** Multivariable-adjusted ORs for COPD in Anhui

	OR (95% CI)	*P* value
Age at survey (years)	1.06 (1.04, 1.08)	0.003
Sex (men)	2.01 (1.22, 3.33)	0.021
Rural residence	1.12 (0.90, 1.40)	0.198
Educational level		
Primary school or lower	1.61 (1.12, 2.31)	0.025
Secondary school	1.07 (0.75, 1.53)	0.582
Higher or further education	1.00(ref)	
Smoking status		
Never smoker	1.00(ref)	
Former smoker	1.15 (0.27, 4.98)	0.775
Current smoker	2.63 (1.86, 3.73)	0.003
Hospital admission for severe pulmonary disease in childhood	0.34 (0.02, 6.43)	0.324
Indoor exposure to biomass for cooking or heating	1.05 (0.65, 1.69)	0.767
Indoor exposure to coal for cooking or heating	1.55 (1.11, 2.15)	0.024
Exposure to dust or chemicals in the workplace	0.85 (0.49, 1.46)	0.402
Family history of lung disease	1.50 (1.17, 1.93)	0.014
History of tuberculosis	1.77 (0.64, 4.84)	0.171
Body-mass index (kg/m^2^)		
< 18.5	0.49 (0.08, 2.96)	0.298
18.5–23.9	1.00(ref)	
24.0–27.9	0.75 (0.34, 1.65)	0.325
≥28.0	0.59 (0.35, 1.01)	0.052

*COPD* chronic obstructive pulmonary disease

## Discussion

This study was conducted in a random sample of the general population aged 40 years or older in Anhui and it followed a stringent quality-control method to improve the validity and reliability of the findings. To the best of our knowledge, this study is the first survey of COPD in Anhui Province, China. As a part of the national survey, the data from this study allows us to provide the direct comparison of COPD prevalence with national estimation in China [6]. The findings also fill several knowledge gaps about the prevalence of COPD in Province. First, the data indicates that 9.8% of the adult population aged 40 years or older had spirometry-defined COPD, which was lower than the national estimation (around 13.6%) in China in 2014–2015 but higher than the previous nationwide estimation (8.2%) in 2002–2004 [5–7]. Second, the proportion (55%) of mild COPD (GOLD I) in Anhui was closed to that (56%) in China in 2014–2015 [6]. However, the proportion was only 24% in the previous survey from 2004 [5]. Third, our investigation demonstrated that 99.6% patients were unaware of their diagnosis in Anhui. Fourth, the percentage (0.7%) of previous lung function examination in identified patients was lower than the percentage of the national survey (5.9%). Fifth, 57.2% patients with COPD were asymptomatic in this study, which was higher than the estimate (35.3%) in 2004 [5]. Different demographic characteristics and exposure levels of COPD-specific risk factors may substantially contribute these differences. For example, mean age (53.8 years in our study and 54.9 years in national survey), proportion of current smoking (28.5% in our study and 31.4% in national survey) and indoor exposure to coal for cooking or heating (28.9% in our study and 34.3% in national survey) were lower than the national survey [6]. In this study, we firstly investigated the prevalence of COPD across regions in one province. The data showed that the north region has a substantively higher risk for developing COPD than south after full adjustments. Thus, differences in genetic and culture background, socioeconomic development level, geographic and climate features in residential areas might also contribute to disparities for COPD prevalence [6].

Tobacco smoking is an established and preventable behavioral factor that contributes to risk of COPD and is very common in Chinese men [19–22]. Although the prevalence of current and former smoking in this survey (36.2%) was lower than that reported in the nationwide survey of COPD (38.4% in 2004 and 40.2% in 2014–2015), the population fraction of COPD attributable to current smoking status was 32.8%, which was higher than national estimation (22.2%) [5, 6]. In this study, we also confirmed that a dose response between COPD prevalence and pack-years is existent (Table 3). Compare to national estimation, COPD is more among subjects who are not exposed to dust and chemicals in the workplace in Anhui population [6]. The results could be attribute to smaller sample size in Anhui and different component proportion of the dust and chemicals in view of different investigated regions. Additionally, biomass use is less associated with prevalence of COPD in women than men in our study as well as in national surveys by using univariate analysis. However, overall association was diminished after full adjustment by using logistic model [6, 7]. Wang et al concluded that heavy outdoor air pollution might mask the effect of biomass fuels [7]. In this study, sex-specific association was also diminished after full adjustments (data not shown). It is worth noting that exposure to indoor air pollution (coal for cooking/heating) could be a novel risk factor for COPD, and the PAF was 12.1% in overall population. We found that indoor exposure to coal has only effect on men in current study. The association was consistent by using fully adjusted model (men: OR = 2.13, *p* = 0.012; women: OR = 0.67, *p* = 0.203). The data suggests that a sex-coal exposure interaction could increase COPD risk in males only. However, this association was not statistically significant in the previous national estimate and further investigation is warranted to validate the effect by prospective study [6].

In the adjusted analysis using logistic model, we also confirmed that male gender, older age at survey and lower educational level were positively correlated with a higher COPD risk [5–7]. Family history of respiratory diseases were also associated with increased prevalence of COPD in Anhui general population which is consistent with previously estimations [6, 7]. In addition, Wang et al found that the association between parental history of respiratory diseases and COPD risk were also existent in never-smokers [7]. We noted that the BMI category of ≥28.0 kg/m^2^ is marginally associated with decreased risk of COPD and COPD is more among subjects with BMI 18.5 to 23.9. These findings might be attributable to residual confounding by smoking status and reverse causality in the cross-sectional study [6]. In addition, the smallest sample size in the BMI category of < 18.5 kg/m^2^ could also limit the estimated precision in this subgroup.

Several limitations of this study should be mentioned. First, oversampled females could cause an underestimate of COPD prevalence since many migrant males were working outside of their permanent residential places. However, the weighted estimations were used to harmonize the sample structure of the study. Second, this cross-sectional survey cannot eliminate recall bias, such as history of tuberculosis or previous COPD diagnosis. Third, people with asthma and other diseases could be misclassified and may result in an overestimate of COPD prevalence [6, 7, 23]. Fourth, exclusion of subjects with severe disease due to the strict rules for spirometry may influence the results [6]. Finally, although the study findings were broadly comparable with the national surveys, the relatively smaller sample size in several subgroups could limit the estimated precision [5–7]. For example, age-specific prevalence of COPD in 60–69 years was slightly decreased in the females.

## Conclusions

In conclusion, our data indicates that COPD is prevalent in the adult population of Anhui Province and the prevalence is highest in north region. The frequency of subjects with COPD who had a previous respiratory function test or who is aware of their diagnosis of COPD are very low. Tobacco smoking and indoor air pollution (exposure to coal for cooking or heating) are major preventable risk factors for the disease in Anhui. Actions such as health promotion for prevention of COPD, early detection of COPD in high-risk individuals, individualized treatment of COPD, and enforcing appropriate region-specific policies are urgently needed to reduce COPD-related burden.

## Additional file

Additional file 1: **Table S1.** American Association for Public Opinion Research outcome rate calculator (Panel of in-person household surveys). **Table S2.** General characteristics of participants included and excluded in the analysis. **Table S3.** Severity of COPD according to GOLD criteria. **Table S4.** Modified MRC dyspnea scale and GOLD ABCD assessment in patients with COPD. **Table S5.** Unweighted prevalence of respiratory symptoms in Patients with COPD in Anhui. **Table S6.** General characteristics of patients with COPD. **Table S7.** Unweighted awareness, diagnosis by spirometry, treatment of COPD among COPD patients. **Table S8.** PAFs for COPD risk factors. (DOC 172 kb)

## Data Availability

The data analyzed in the current study are not publicly available but may be made available from the corresponding authors on reasonable request.

## Abbreviations

BMI: Body-mass index
COPD: Chronic obstructive pulmonary disease
DSP: Disease surveillance point
FEV1: Forced expiratory volume in 1 s
FVC: Forced vital capacity (FVC)
GOLD: The Global Initiative for Chronic Obstructive Lung Disease
LLN: Lower limits of normal
mMRC: the modified Medical Research Council
PAF: Population-attributable fraction
STROBE: the Strengthening the Reporting of Observational Studies in Epidemiology
