# Supplementary material for: Prevalence and risk factors of chronic obstructive pulmonary disease in Anhui Province, China: a population-based survey
Source: BMC Pulm Med. 2019 May 29;19:102. doi: 10.1186/s12890-019-0864-0 (PMC6542059; doi:10.1186/s12890-019-0864-0)
Supplement: Supplementary file 1 — Table S1. American Association for Public Opinion Research outcome rate calculator (Panel of in-person household surveys). Table S2. General characteristics of participants included and excluded in the analysis. Table S3. Severity of COPD according to GOLD criteria. Table S4. Modified MRC dyspnea scale and GOLD ABCD assessment in patients with COPD. Table S5. Unweighted prevalence of respiratory symptoms in Patients with COPD in Anhui. Table S6. General characteristics of patients with COPD. Table S7. Unweighted awareness, diagnosis by spirometry, treatment of COPD among COPD patients. Table S8. PAFs for COPD risk factors. (DOC 172 kb) [file 12890_2019_864_MOESM1_ESM.doc]

**SUPPLEMENTARY INFORMATION**

**Prevalence and Risk Factors of Chronic Obstructive Pulmonary Disease in Anhui Province, China: A Population-based Survey**

Zhenqiu Zha^1,2*^, Ruixue Leng^1*^, Wei Xu^2^, Heling Bao^3^, Yeji Chen^2^, Liwen Fang^3^, Zhirong Liu^2^ and Dongqing Ye^1,4^

^1^Department of Epidemiology and Biostatistics, School of Public Health, Anhui Medical University, Hefei, Anhui, China

^2^Anhui Provincial center for Disease Control and Prevention, Anhui, China

^3^National Center for Chronic and Non-Communicable Disease Control and Prevention, Chinese Center for Disease Control and Prevention, Beijing, China

^4^Clinic Medical College of Anhui Medical University, Hefei, Anhui, China

**Supplementary Tables**

Table S1. American Association for Public Opinion Research outcome rate calculator (Panel of in-person household surveys).

Table S2. General characteristics of participants included and excluded in the analysis.

Table S3. Severity of COPD according to GOLD criteria.

Table S4. Modified MRC dyspnea scale and GOLD ABCD assessment in patients with COPD.

Table S5. Unweighted prevalence of respiratory symptoms in Patients with COPD in Anhui.

Table S6. General characteristics of patients with COPD.

Table S7. Unweighted awareness, diagnosis by spirometry, treatment of COPD among COPD patients.

Table S8. PAFs for COPD risk factors.

**Table S1.** American Association for Public Opinion Research outcome rate calculator (Panel of in-person household surveys).

|  | Final Disposition Codes^†^ | COPD survey in 2015^†^ |
| --- | --- | --- |
| Interview (Category 1) | 1.0 |  |
| Complete | 1.1 | 2840 |
| Partial | 1.2 | 156 |
| Eligible, non-interview (Category 2) | 2.0 |  |
| Refusal and breakoffs | 2.10 | 4 |
| Non-contact | 2.20 | 0 |
| Other, non-refusals | 2.30 | 0 |
| Total sample used |  | 3000 |
| I=Complete Interviews (1.1) |  | 2840 |
| P=Partial Interviews (1.2) |  | 156 |
| R=Refusal and break off (2.10) |  | 4 |
| NC=Non-contact (2.20) |  | 0 |
| O=Other (2.30) |  | 0 |
| Response Rate 1 |  |  |
| I/((I+P) + (R+NC+O) + (UH+UO)) |  | 0.947 |
| Response Rate 2 |  |  |
| (I+P)/((I+P) + (R+NC+O) + (UH+UO)) |  | 0.999 |
| Response Rate 3 |  |  |
| I/((I+P) + (R+NC+O) + e(UH+UO)) |  | 0.947 |
| Response Rate 4 |  |  |
| (I+P)/((I+P) + (R+NC+O) + e(UH+UO)) |  | 0.999 |
| Cooperation Rate 1 |  |  |
| I/((I+P)+R+O)) |  | 0.947 |
| Cooperation Rate 2 |  |  |
| (I+P)/((I+P)+R+O)) |  | 0.999 |
| Cooperation Rate 3 |  |  |
| I/((I+P)+R)) |  | 0.947 |
| Cooperation Rate 4 |  |  |
| (I+P)/((I+P)+R)) |  | 0.999 |
| Refusal Rate 1 |  |  |
| R/((I+P)+(R+NC+O) + UH + UO)) |  | 0.001 |
| Refusal Rate 2 |  |  |
| R/((I+P)+(R+NC+O) + e(UH + UO)) |  | 0.001 |
| Refusal Rate 3 |  |  |
| R/((I+P)+(R+NC+O)) |  | 0.001 |
| Contact Rate 1 |  |  |
| ((I+P)+R+O)/ ((I+P)+R+O+NC+ (UH + UO)) |  | 1.000 |
| Contact Rate 2 |  |  |
| ((I+P)+R+O) / ((I+P)+R+O+NC + e(UH+UO)) |  | 1.000 |
| Contact Rate 3 |  |  |
| ((I+P)+R+O) / ((I+P)+R+O+NC) |  | 1.000 |

^†^ Contents listed in the original table but not applicable for this survey were not listed.

**Table S2.** General characteristics of participants included and excluded in the analysis.

| Groups | Subjects excluded | Subjects included | *P* value^†^ |
| --- | --- | --- | --- |
| Total | 226 | 2770 |  |
| Age group |  |  |  |
| 40-49 years | 48(21.2) | 1047(37.8) | <0.001 |
| 50-59 years | 55(24.3) | 788(28.5) |  |
| 60-69 years | 63(27.9) | 635(22.9) |  |
| ≥70 years | 60(26.6) | 300(10.8) |  |
| Sex |  |  |  |
| Male | 129(57.1) | 1362(49.2) | 0.022 |
| Female | 97(42.9) | 1408(50.8) |  |
| Residence |  |  |  |
| Urban | 131(58.0) | 1668(60.2) | 0.506 |
| Rural | 95(42.0) | 1102(39.8) |  |
| Educational level |  |  |  |
| Primary school or lower | 136(60.2) | 1690(61.0) | 0.227 |
| Secondary school | 57(25.2) | 774(28.0) |  |
| Higher or further education | 33(14.6) | 305(11.0) |  |
| Smoking status |  |  |  |
| Never smoker | 138(61.1) | 1758(63.3) | 0.366 |
| Former smoker | 25(11.1) | 231(8.6) |  |
| Current smoker | 63(27.9) | 780(28.2) |  |
| Hospital admissions due to severe pulmonary diseases in childhood | | | |
| Yes | 4(1.8) | 53(1.9) | 0.879 |
| No | 222(98.2) | 2716(98.1) |  |
| Indoor exposure to biomass for cooking or heating | | | |
| Yes | 92(40.7) | 961(34.7) | 0.069 |
| No | 134(59.3) | 1808(65.3) |  |
| Indoor exposure to coal for cooking or heating | | | |
| Yes | 27(12.0) | 518(18.7) | 0.011 |
| No | 199(88.1) | 2251(81.3) |  |
| Exposure to dust or chemical at the workplace | | | |
| Yes | 77(34.1) | 1152(41.6) | 0.027 |
| No | 149(66.0) | 1617(58.4) |  |
| History of tuberculosis | | | |
| Yes | 5(2.2) | 46(1.7) | 0.538 |
| No | 221(97.8) | 2723(98.3) |  |

^†^ Unweighted estimations were used.

**Table S3. Severity of COPD according to GOLD criteria.**

|  | Overall (n=269) ^†^ | | Men (n=208) | | Women (n=61) | |
| --- | --- | --- | --- | --- | --- | --- |
|  | Cases (n) | Proportion (95% CI) | Cases (n) | Proportion (95% CI) | Cases (n) | Proportion (95% CI) |
| GOLD stage I (mild) | 148 | 55.0% (49.0, 60.9) | 105 | 50.5% (43.7, 57.3) | 43 | 70.5% (57.8, 80.7) |
| GOLD stage II (moderate) | 95 | 35.3% (29.8, 41.3) | 79 | 38.0% (31.6, 44.8) | 16 | 26.2% (16.6, 38.8) |
| GOLD stage III/IV (severe or very severe) | 26 | 9.7% (6.6, 13.8) | 24 | 11.5% (7.8, 16.7) | 2 | 3.3% (0.8, 12.4) |

^†^ Three patients were excluded due to missing information for predicted values of FEV1(only available for 40-81years).

COPD, chronic obstructive pulmonary disease; GOLD, Global Initiative for Chronic Obstructive Lung Disease.

**Table S4.** Modified MRC dyspnea scale and GOLD ABCD assessment in patients with COPD.

| Groups | Overall | | Men | | Women | |
| --- | --- | --- | --- | --- | --- | --- |
|  | No. of patients^†^ | % (95% CI) | No. of patients | % (95% CI) | No. of patients | % (95% CI) |
| Modified MRC dyspnea scale |  |  |  |  |  |  |
| Grade 0 | 229 | 84.8(80.0, 88.6) | 176 | 84.2(78.6, 88.6) | 53 | 86.9(75.5, 93.4) |
| Grade 1 | 36 | 13.3(9.6, 18.0) | 28 | 13.4(9.4, 18.8) | 8 | 13.1(6.6, 24.5) |
| Grade 2 | 2 | 0.7(0.2, 2.9) | 2 | 1.0(0.2, 3.8) | 0 | - |
| Grade 3 | 3 | 1.1(0.4, 3.4) | 3 | 1.4(0.5, 4.4) | 0 | - |
| Grade 4 | 0 | - | 0 | - | 0 | - |
| GOLD ABCD assessment |  |  |  |  |  |  |
| GOLD A | 253 | 93.7(90.1, 96.1) | 195 | 93.3(89.0, 96.0) | 58 | 95.1(85.4, 98.5) |
| GOLD B | 5 | 1.9(0.8, 4.4) | 5 | 2.4(1.0, 5.7) | 0 | - |
| GOLD C | 12 | 4.4(2.5, 7.7) | 9 | 4.3(2.2, 8.1) | 3 | 4.9(1.5, 14.6) |
| GOLD D | 0 | - | 0 | - | 0 | - |

^†^ Total 270 COPD patients were included for mMRC dyspnea scale and GOLD ABCD assessment. Two patients were excluded due to missing information.

COPD, chronic obstructive pulmonary disease; GOLD, Global Initiative for Chronic Obstructive Lung Disease; mMRC, the modified Medical Research Council.

**Table S5.** Unweighted prevalence of respiratory symptoms in Patients with COPD in Anhui.

|  | No. of Participants | % (95% CI) | | | | | |
| --- | --- | --- | --- | --- | --- | --- | --- |
|  |  | Cough | Sputum | Wheezing | Dyspnea | One of above | Chronic cough and phlegm |
| All COPD patients ^†^ | 271 | 17.34(13.3, 22.4) | 20.6(16.2-25.9) | 21.8(17.2, 27.1) | 24.0(19.2, 29.5) | 42.8(37.0, 48.8) | 15.1(11.3, 19.9) |
| Age group ^†^ |  |  |  |  |  |  |  |
| 40-49 years | 41 | 9.8(3.6, 24.1) | 12.2(5.0, 26.9) | 12.2(5.0, 26.9) | 12.2(5.0, 26.9) | 31.7(18.9, 48.0) | 7.3(2.3, 21.2) |
| 50-59 years | 64 | 18.8(10.8, 30.5) | 18.8(10.8, 30.5) | 20.3(12.0, 32.3) | 17.2(9.6, 28.8) | 39.1(27.7, 51.8) | 12.5(6.3, 23.4) |
| 60-69 years | 100 | 17.0(10.8, 25.8) | 23.0(15.7, 32.4) | 23.0(15.7, 32.4) | 27.0(19.1, 36.7) | 48.0(38.3, 57.9) | 16.0(10.0, 24.7) |
| ≥70 years | 66 | 21.2(12.8, 33.0) | 24.2(15.2, 36.3) | 27.3(17.7, 39.5) | 33.3(22.8, 45.8) | 45.5(33.6, 57.8) | 21.2(12.8, 33.0) |
| *P* value for trend |  | 0.207 | 0.114 | 0.068 | 0.004 | 0.104 | 0.040 |
| Sex ^†^ |  |  |  |  |  |  |  |
| Male | 209 | 18.7(13.9, 24.6) | 23.4(18.1, 29.7) | 23.4(18.2, 29.7) | 24.9(19.4, 31.2) | 46.9(40.2, 53.7) | 16.3(11.8, 22.0) |
| Female | 62 | 12.9(6.5, 24.1) | 11.3(5.4, 22.2) | 16.1(8.7, 27.8) | 21.1(12.4, 33.2) | 29.0(18.9, 41.8) | 11.3(5.4, 22.2) |
| *P* value |  | 0.293 | 0.038 | 0.220 | 0.526 | 0.013 | 0.337 |
| GOLD stages ^‡^ |  |  |  |  |  |  |  |
| GOLD I | 147 | 12.2(7.8, 18.7) | 17.7(12.3, 24.8) | 13.6(8.9, 20.2) | 15.6(10.6, 22.5) | 32.7(25.5, 40.7) | 11.6(7.3, 17.9) |
| GOLD II | 95 | 21.1(13.9, 30.6) | 18.9(12.2, 28.3) | 28.4(20.1, 38.5) | 26.3(18.3, 36.2) | 50.5(40.4, 60.6) | 15.8(9.7, 24.7) |
| GOLD III/IV | 26 | 30.8(15.4, 52.0) | 42.3(24.2, 62.8) | 42.3(24.1, 62.8) | 61.5(40.7, 78.9) | 73.1(51.7, 87.3) | 30.8(15.4, 52.0) |
| *P* value for trend |  | 0.009 | 0.026 | <0.001 | <0.001 | <0.001 | 0.020 |

^†^ One patient was excluded due to missing information for respiratory symptoms.

^‡^ One patient was excluded due to missing information for respiratory symptoms, and 3 patients were further excluded due to missing information for predicted values of FEV1(only available for 40-81years).

COPD, chronic obstructive pulmonary disease; GOLD, Global Initiative for Chronic Obstructive Lung Disease.

**Table S6.** General characteristics of patients with COPD.

|  | % (95% CI) | | | |
| --- | --- | --- | --- | --- |
|  | Overall (n=271) ^†^ | GOLD I (n=147) ^‡^ | GOLD II (n=95) ^‡^ | GOLD III/IV (n=26) ^‡^ |
| History of tuberculosis (Yes) | 3.3(1.7, 6.3) | 2.0(0.7, 6.2) | 3.2(1.0, 9.5) | 11.5(3.5, 32.1) |
| Past year exacerbation (Yes) | 5.9(3.6, 9.4) | 2.7(1.0, 7.1) | 8.4(4.2, 16.1) | 15.4(5.5, 36.3) |
| Past year hospitalization (Yes) | 1.8(0.7, 4.4) | 0.7(0.1, 4.8) | 3.2(1.0, 9.5) | 3.8(0.5, 25.4) |
| Co-morbidities (Yes) |  |  |  |  |
| Pulmonary heart disease | 0.4(0.1, 2.6) | 0 | 1.1(0.1, 7.3) | 0 |
| Coronary heart disease | 4.4(2.5, 7.7) | 2.7(1.0, 7.1) | 7.4(3.5, 14.8) | 3.8(0.5, 25.4) |
| Diabetes | 2.6(1.2, 5.3) | 1.4(0.3, 5.3) | 4.2(1.6, 10.9) | 3.8(0.5, 25.4) |
| Hypertension | 25.8(20.9, 31.4) | 23.8(17.5, 31.5) | 28.4(20.1, 38.5) | 26.9(12.7, 48.3) |
| Depression | 0 | 0 | 0 | 0 |
| Osteoporosis | 1.8(0.8, 4.4) | 1.4(0.3, 5.4) | 2.1(0.5, 8.2) | 3.8(0.5, 25.4) |

Note: The prevalence of co-morbidities was calculated based on self-report data from questionnaires.

^†^ One patient was excluded due to missing information for characteristics in overall population.

^‡^ One patient was excluded due to missing information for respiratory symptoms, and 3 patients were further excluded due to missing information for predicted values of FEV1(only available for 40-81years).

COPD, chronic obstructive pulmonary disease; GOLD, Global Initiative for Chronic Obstructive Lung Disease.

**Table S7.** Unweighted awareness, diagnosis by spirometry, treatment of COPD among COPD patients.

|  | Awareness | | Suspected COPD by Spirometry | | Treatment of COPD | |
| --- | --- | --- | --- | --- | --- | --- |
| Groups | No. of Participants^†^ | % (95% CI) | No. of Participants^†^ | % (95% CI) | No. of Participants^†^ | % (95% CI) |
| Overall | 271 | 0.4(0.1, 2.6) | 271 | 0.7(0.2, 2.9) | 271 | 7.7(5.1, 11.6) |
| Age group |  |  |  |  |  |  |
| 40-49 years | 41 | 0 | 41 | 0 | 41 | 4.9(1.2, 17.9) |
| 50-59 years | 64 | 0 | 64 | 0 | 64 | 3.1(0.8, 11.8) |
| 60-69 years | 100 | 1.0(0.1, 6.9) | 100 | 1.0(0.1, 6.9) | 100 | 6.0(2.7, 12.8) |
| ≥70 years | 66 | 0 | 66 | 1.5(0.2, 10.2) | 66 | 16.7(9.4, 27.8) |
| *P* value for trend |  | - |  | - |  | 0.011 |
| Sex |  |  |  |  |  |  |
| Male | 209 | 0.5(0.1, 3.4) | 209 | 1.0(0.2, 3.8) | 209 | 8.6(5.5, 13.3) |
| Female | 62 | 0 | 62 | 0 | 62 | 4.8(1.5, 14.1) |
| *P* value |  | - |  | - |  | 0.329 |

^†^ One patient was excluded due to missing information. COPD, chronic obstructive pulmonary disease.

**Table S8. PAFs for COPD risk factors.**

|  | OR (95% CI)^‡^ | Overall | | Men | | Women | |
| --- | --- | --- | --- | --- | --- | --- | --- |
|  |  | Pe (95% CI) ^†^ | PAF (95% CI) | Pe (95% CI) ^†^ | PAF (95% CI) | Pe (95% CI) ^†^ | PAF (95% CI) |
| Current smoker | 2.63(1.86, 3.73) | 52.9% (34.6, 70.4) | 32.8% (25.4, 39.4) | 70.9% (43.6, 88.5) | 44.0% (33.8, 52.6) | 6.3% (1.4, 24.5) | 3.9% (3.1, 4.7) |
| Indoor exposure to coal | 1.55(1.11, 2.15) | 34.0% (5.6, 81.8) | 12.1% (4.5, 19.0) | 38.1% (7.1, 83.3) | 13.5% (5.0, 21.3) | 23.4% (2.1, 81.2) | 8.3% (3.2, 13.2) |

^†^ Pe is the exposure prevalence among cases.

^‡^ The adjusted OR from multivariate logistic model in the study (as shown in Table 4). Only modifiable risk factors with significant ORs were presented.

COPD, chronic obstructive pulmonary disease; PAF, population-attributable fraction.
